# Real-world hospitalization and readmission trajectory after Guillain-Barré syndrome in the United States

**DOI:** 10.1186/s12883-026-05499-9

**Published:** 2026-10-22

**Authors:** Jan Voth, Julius Gerstmeyer, Sven G. Meuth, Gerd Meyer zu Hörste, Alice Grizzel Willison, Franz Felix Konen, Hugh John Willison, Thomas Skripuletz, Michael Gliem, Marc Pawlitzki, Jens Chapman, Lars Masanneck

**Affiliations:** 1 Department of Neurology, Medical Faculty, University Hospital Düsseldorf, Heinrich Heine University Düsseldorf, Düsseldorf, Germany; 2 Department of Orthopedics and Trauma Surgery, St. Josef Hospital Bochum, Bochum, Germany; 3 Department of Neurology, University Hospital Münster, Münster, Germany; 4 Department of Neurology, Hannover Medical School, Hannover, Germany; 5 College of Medicine, Veterinary and Life Sciences, University of Glasgow, Glasgow, UK; 6 Swedish Neuroscience Institute, Swedish Medical Center, Seattle, WA USA; 7 Digital Engineering Faculty, Hasso Plattner Institute, University of Potsdam, Potsdam, Germany

**Keywords:** Guillain-Barré syndrome, Hospital readmission, Nationwide Readmissions Database, Treatment coding, Post-acute care

## Abstract

**Background:**

Guillain-Barré syndrome (GBS) is an acute immune-mediated polyradiculoneuropathy associated with substantial morbidity, but contemporary data on post-discharge readmission remain limited. We characterized 90-day readmission timing, causes, associated factors, and procedure coding after GBS.

**Methods:**

We analyzed an unweighted cohort of adults with a principal diagnosis of GBS in the 2020 Nationwide Readmissions Database. The primary outcome was all-cause readmission within 90 days after discharge. Readmission causes were classified using principal readmission diagnoses mapped to Clinical Classifications Software Refined categories, and procedure coding was descriptively assessed using ICD-10 procedure codes and procedure-day variables. Logistic regression models were used to identify factors associated with readmission.

**Results:**

Among 2,587 adults eligible for 90-day follow-up, 364 (14.1%) were readmitted. Readmissions clustered early, with 170 (46.7%) occurring within 15 days and 241 (66.2%) within 30 days. Separately, GBS was the principal diagnosis in 170 of 364 readmissions (46.7%), representing the largest grouped cause followed by septicemia (9.6%) and cardiovascular or thromboembolic conditions (6.9%). In adjusted analyses, discharge disposition was the most consistent marker of readmission risk: compared with routine home discharge, facility discharge was associated with higher odds of 90-day readmission (odds ratio 1.70, 95% confidence interval 1.27–2.28). Procedure-code timing suggested that diagnostic evaluation and immunotherapy were administered early, whereas plasma exchange, tracheostomy, gastrostomy, and rehabilitation-related procedures were performed at later stages.

**Conclusions:**

Readmission after GBS is frequent, early, and clinically heterogeneous, supporting its interpretation as a marker of residual neurological disability, systemic complications, and care-transition vulnerability rather than recurrent GBS alone.

**Supplementary Information:**

The online version contains supplementary material available at https://doi.org/10.1186/s12883-026-05499-9.

## Background

Guillain-Barré syndrome (GBS) is a rare, acute, immune-mediated polyradiculoneuropathy and one of the leading causes of acute flaccid paralysis worldwide [1]. It is typically characterized by ascending symmetrical weakness, areflexia, and frequent involvement of autonomic pathways [1, 2]. 

Although GBS is classically monophasic, its acute course is often severe. A substantial proportion of patients require intensive care due to respiratory failure, autonomic instability, or rapidly progressive weakness. Up to 30% of patients require mechanical ventilation, and acute-phase mortality remains clinically relevant [3–5]. Severe cases may require a tracheostomy or extended inpatient care and are often accompanied by complications such as pneumonia, sepsis, and prolonged functional dependence [4, 6]. 

Established immunotherapy is currently limited to intravenous immunoglobulin (IVIG) and plasma exchange (PLEX) [7]. Despite treatment, recovery is frequently incomplete at discharge. Residual weakness, impaired walking ability, fatigue, and the need for post-acute rehabilitation are common, emphasizing a clinical burden of GBS beyond the index hospitalization [1, 4]. 

Post-discharge vulnerability after GBS is likely heterogeneous. Possible causes may reflect complications of severe acute disease and immobility, including respiratory infection, thromboembolism, or cardiovascular instability. Others may reflect disease-intrinsic mechanisms, including treatment-related fluctuation (TRF), defined as recurrent deterioration after initial improvement or stabilization [8], or diagnostic reclassification in patients with acute-onset chronic inflammatory demyelinating polyradiculoneuropathy (CIDP) [9]. 

Hospital readmission can therefore be used as a marker of post-acute disease burden, care transitions, and unresolved clinical vulnerability after hospitalization [10–12]. Additionally, across diseases, readmission is associated with poorer subsequent outcomes, including increased mortality, disability, and reduced quality of life. In GBS, contemporary readmission data remain limited, with prior work using 2013 NRD-based data reporting substantial 30-day (12.2%) and 1-year (20.8%) readmission rates and identifying respiratory failure, heart failure, and renal failure as associated factors [13]. However, contemporary International Classification of Diseases, Tenth Revision (ICD-10)-era data describing the timing of readmission, cause profile, and treatment coding patterns remain missing.

Using an unweighted cohort derived from the 2020 Nationwide Readmissions Database (NRD), we aimed to characterize hospital readmission after index GBS hospitalization in a large US hospital-based cohort. Specifically, we aimed to describe timing and causes of 90-day readmissions, to identify adjusted associations of readmission, and to characterize treatment and procedure coding patterns relevant to acute and post-acute GBS care.

## Methods

### Study design and data source

We conducted a retrospective cohort study using unweighted data extracted from the 2020 NRD. The NRD is part of the Healthcare Cost and Utilization Project (HCUP), by the Agency for Healthcare Research and Quality, and was developed to support analyses of hospital readmissions within a calendar year in the United States [14]. The database contains discharge-level inpatient records with patient linkage identifiers, timing variables, diagnosis and procedure codes [14]. The 2020 NRD was constructed from 31 geographically dispersed HCUP Partner States, representing 62.2% of the US resident population and 60.8% of US hospitalizations [14]. State identifiers were not available in the NRD-derived analytic extract, precluding state-specific analyses. Since discharge weights were not available either in the dataset, all analyses were conducted unweighted and should be interpreted as findings within the cohort rather than nationally representative estimates.

### Study population

We included adult hospitalizations, defined as patient age ≥ 18 years, with GBS as the principal reason for admission, identified by ICD-10-CM code G61.0 in the first diagnosis position. Admissions were required to contain valid NRD linkage and timing variables. Index admissions ending in in-hospital death were excluded. For patients with more than one qualifying GBS hospitalization, the first qualifying GBS hospitalization was selected as the index admission. Because the primary analysis focused on 90-day readmission, index discharges were restricted to January through September to ensure a complete 90-day follow-up window within the NRD calendar year. To assess potential calendar-related selection arising from this cohort restriction baseline characteristics of this cohort were compared with those of patients discharged from October through December. Balance was assessed using absolute standardized mean differences, with values ≥ 0.10 considered indicative of potentially meaningful imbalance.

A secondary 30-day analysis was performed to facilitate comparison with prior NRD-based GBS readmission data. For this analysis, the eligible discharge window was extended to January through November, as this window provides complete 30-day follow-up while maximizing sample size and facilitating comparison with the prior NRD-based readmission study [13]. 

### Readmission outcomes, timing and cause classification

The primary outcome was all-cause readmission within 90 days after discharge from the index GBS hospitalization. Readmission was defined as the first subsequent inpatient admission for the same linked patient within the respective post-discharge window. Readmission timing was calculated from index discharge to subsequent admission using NRD timing variables [15]. Prespecified timing intervals were 1–15, 16–30, 31–60, and 61–90 days after discharge. For early-versus-late comparisons, readmissions within the 90-day eligible cohort were grouped as days 1–30 versus days 31–90. For a descriptive comparison of readmissions among patients receiving immunotherapy, immunotherapy was defined as IVIG and/or PLEX administration.

Causes of readmission were defined using the principal diagnosis of the first readmission within the 90-day window, subsequent readmissions were not considered. Principal readmission diagnoses were mapped to HCUP Clinical Classifications Software Refined (CCSR) categories [16]. The polyneuropathy CCSR category was further separated into GBS, CIDP, and other polyneuropathy diagnoses using ICD-10-CM codes. A 30-day readmission analysis was performed as a secondary analysis.

For exploratory paired severity analyses among patients with an eligible first 90-day readmission, index-admission characteristics were compared with the corresponding readmission hospitalization within the same patient. Paired measures, including length of stay (LOS) and All Patient Refined Diagnosis Related Group (APR-DRG) severity, were compared descriptively and using Wilcoxon signed-rank tests.

In a post hoc sensitivity analysis, we assessed the ability of index-hospitalization LOS to discriminate between patients with and without 90-day readmission using receiver operating characteristic analysis. The LOS threshold maximizing the Youden index was identified, and its sensitivity, specificity, positive predictive value (PPV), and negative predictive value (NPV) were calculated. The 95% confidence interval for the AUROC was estimated using 5,000 bootstrap samples.

### Regression analyses of readmission markers

Regression analyses were performed in the 90-day readmission cohort. To identify markers associated with readmission, we used logistic regression models and reported odds ratios with 95% confidence intervals. To account for within-hospital correlation, confidence intervals and p values were calculated using cluster-robust covariance estimates grouped by the hospital of the index admission (HOSP_NRD). First, unadjusted models were fitted for each candidate variable or categorical variable group to describe crude associations with 90-day readmission.

We then fitted an adjusted multivariable logistic regression model to examine associations after accounting for clinically relevant covariates. The multivariable model was intended to describe adjusted associations between discharge-time markers and readmission. It included 27 non-intercept coefficients. Variables were selected based on clinical relevance, previous readmission literature, and availability within the database and were grouped into clinically interpretable domains. Demographic and socioeconomic variables included age, sex, payer group, and ZIP-code income quartile. Index-admission severity was represented by LOS, APR-DRG severity, and recording of respiratory failure. Recorded acute immunotherapy during the index admission was modeled categorically as IVIG-only therapy or PLEX-containing therapy, each compared with neither IVIG nor PLEX. Discharge disposition was modeled as a mutually exclusive categorical variable, with facility discharge, home health care, short-term hospital transfer, and other discharge destinations compared with routine home discharge. Comorbidities and complication-related factors were included as additional adjustment variables. Missing payer and discharge disposition were retained in the “other” or “other/missing” categories. Multicollinearity was assessed using variance inflation factors. Apparent model discrimination was evaluated using the area under the receiver operating characteristic curve (AUROC), and overall prediction error using the Brier score.

Two sensitivity analyses were performed to assess the robustness of the primary 90-day model. First, because treatment exposure was based on procedure coding and may be incompletely captured, the model was repeated after excluding the treatment category while retaining the same analytic sample, covariates, and hospital-clustered standard errors. Second, to account more extensively for markers of index-admission severity and supportive care, mechanical ventilation, tracheostomy, gastrostomy, and rehabilitation procedures were additionally included, again retaining hospital-clustered standard errors. As a secondary analysis, the same structured modeling approach was repeated for 30-day readmission to facilitate comparison with the commonly used early readmission window and prior NRD-based GBS data. To explore whether different factors were associated with the timing of readmission, an additional readmitted-only model compared early readmission, defined as days 1–30, with later readmission, defined as days 31–90. This timing model was restricted to patients who were readmitted within 90 days.

### Timing of treatment and procedure coding

For the exploratory treatment-pathway analysis, index treatment and procedure indicators were derived from ICD-10-PCS procedure-code fields. PLEX and mechanical ventilation were identified using predefined ICD-10-PCS code sets. IVIG was approximated using specific globulin-administration procedure codes. Procedure timing was assessed using procedure-day variables and PRCCSR categories and expressed relative to the index admission date. Timing distributions were visualized in a row-normalized heatmap to describe when specific procedure groups were recorded during the index hospitalization. Patients could contribute to multiple intervals for the same procedure. In a separate first-procedure analysis, only the earliest recorded day for each procedure per patient was used. This analysis was descriptive and should not be interpreted as a validated treatment sequence.

### Statistical analysis

Statistical analyses were performed in Python 3.11.9 using pandas, numpy, scipy, and statsmodels. Normally distributed continuous variables were reported as mean and standard deviation (SD), non-normally distributed variables as median and interquartile range (IQR), and categorical variables as counts and percentages. For grouped readmission-cause comparisons, global cause distributions were compared using a chi-square framework; Monte Carlo permutation p values were used when expected cell counts were sparse. Cause-specific early-versus-late comparisons used Fisher exact tests. Where multiple comparisons were performed, p values were adjusted using the Benjamini-Hochberg false-discovery-rate (FDR) procedure and reported as q values. In accordance with HCUP reporting requirements, cell counts of 10 or fewer were suppressed in tables, figures, and display outputs.

## Results

### Cohort selection and readmission burden

**Fig. 1:**
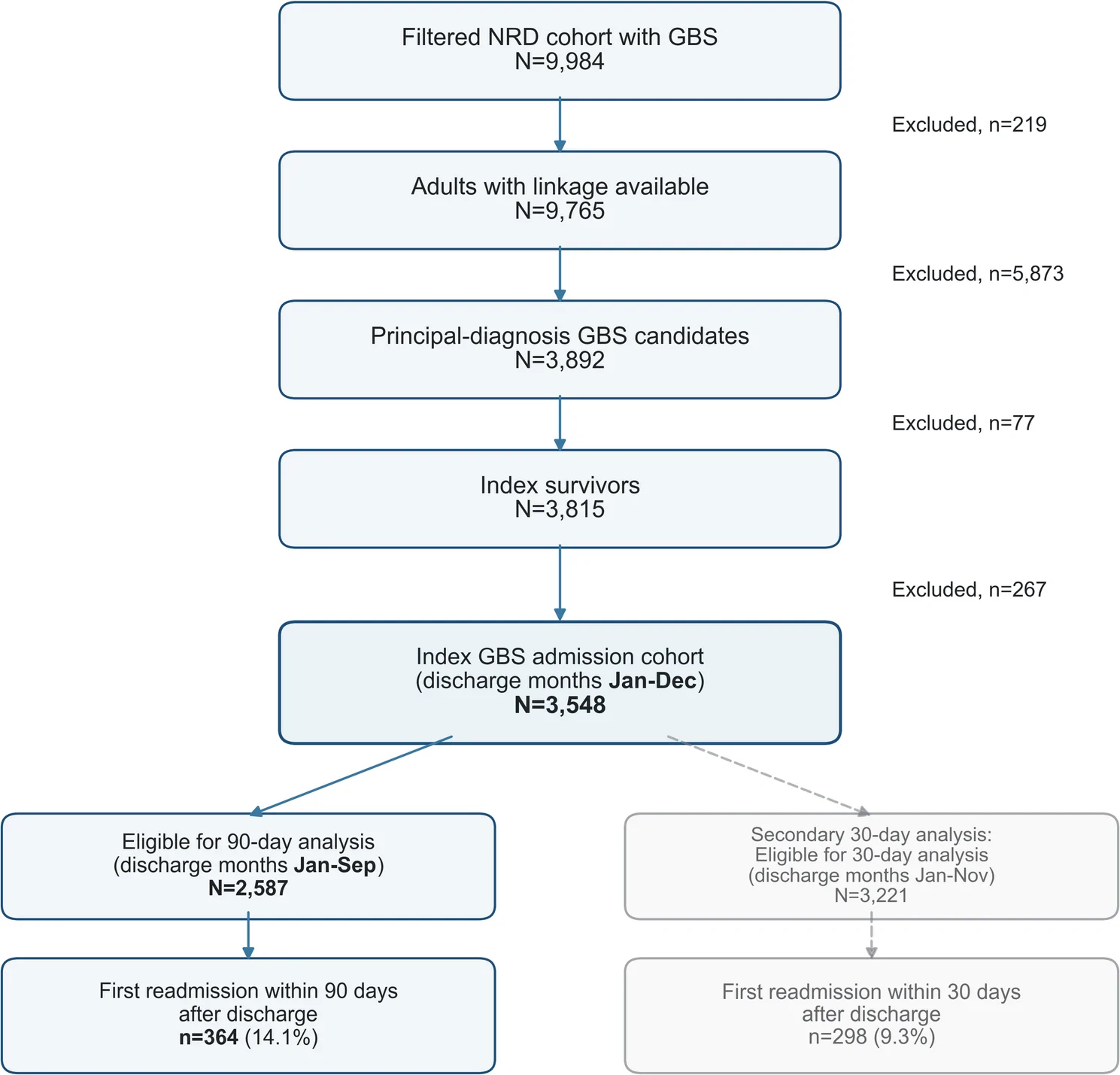
Study cohort selection. Flow diagram showing the sequential identification of adult index admissions with a principal diagnosis of Guillain-Barré syndrome. Admissions without the required patient-linkage information and patients who died during the index hospitalization were excluded. For patients with multiple qualifying admissions, only the first admission was retained as the index admission. The final index-admission cohort comprised 3,548 patients discharged from January through December. The primary 90-day analysis included 2,587 patients discharged from January through September with sufficient calendar-year follow-up, of whom 364 (14.1%) experienced a first readmission within 90 days after discharge. The faded branch shows the secondary 30-day analysis, which included 3,221 patients discharged from January through November, of whom 298 (9.3%) experienced a first readmission within 30 days after discharge. Abbreviations: GBS, Guillain-Barré syndrome; NRD, Nationwide Readmissions Database

The source dataset contained 9,984 hospitalization records. After cohort filtering, the final index cohort comprised 3,548 adults with a first GBS admission between January and December 2020. Baseline characteristics are provided in Suppl. Table 1. Of these patients, 2,587 were discharged between January and September and were included in the primary 90-day readmission analysis. Among them, 364 (14.1%) were readmitted within 90 days after discharge (Fig. 1). Among patients with a 90-day readmission, readmission hospitalizations were slightly shorter than index admissions. Median LOS decreased from 9 days (IQR 5–18) during the index admission to 8 days (IQR 4–16) at readmission, with a median paired change of -1 day (IQR − 7 to 4, Wilcoxon *p* = 0.006). Despite the modestly shorter LOS, administrative severity increased on readmission. Median APR-DRG severity increased from 2 (IQR 2–3) at the index admission to 3 (IQR 2–4) at readmission (Wilcoxon *p* < 0.001; Suppl. Table 2). Cause-specific patterns are summarized in Suppl. Table 3.

Baseline characteristics of the 2,587 patients included in the primary 90-day analysis and the 961 patients excluded because of insufficient calendar-year follow-up are compared in Suppl. Table 4. The excluded patients were modestly older and more frequently had Medicare coverage, diabetes, acute kidney injury, greater APR-DRG severity, and non-routine-home discharge. Other characteristics were generally similar.

For the secondary 30-day readmission analysis, 3,221 patients were eligible (discharge January to November), of whom 298 (9.3%) were readmitted within 30 days.

### Readmissions clustered in the first 30 days after discharge

**Fig. 2:**
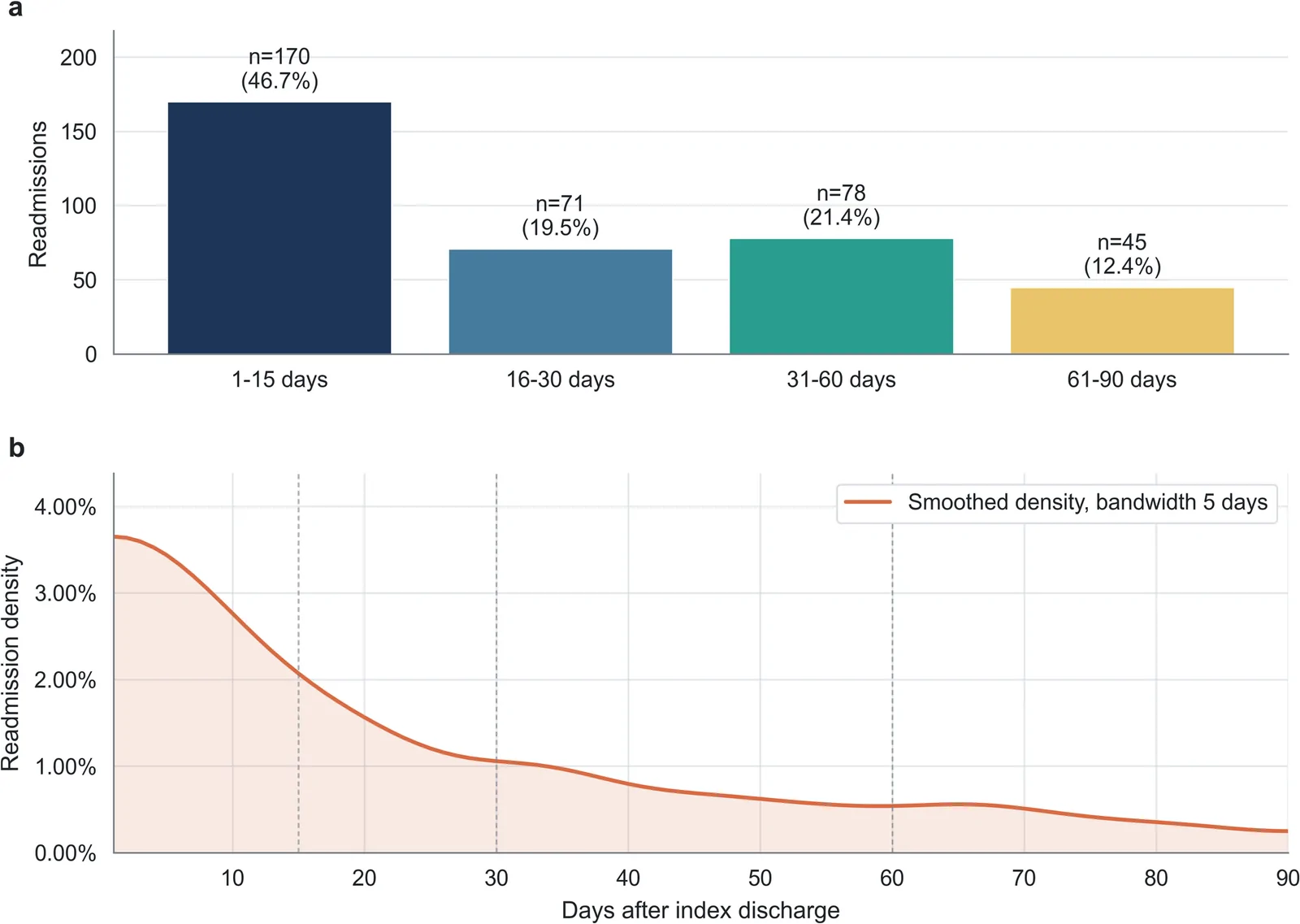
Timing of first readmission within 90 days after discharge. The analysis included 364 patients with a first readmission among the 2,587 patients eligible for 90-day follow-up. **a** Number and proportion of first readmissions occurring at 1–15, 16–30, 31–60, and 61–90 days after discharge. Percentages were calculated using all first 90-day readmissions as the denominator. **b** Kernel-smoothed empirical distribution of the time from index discharge to first readmission, using a bandwidth of 5 days. Dashed vertical lines indicate the boundaries at days 15, 30, and 60. The median time to readmission was 18 days (interquartile range, 7–39 days). The smoothed curve is descriptive and does not represent a hazard function or model-based risk estimate

Readmissions were concentrated early after discharge. Among 90-day readmissions, 170 of 364 (46.7%) occurred during days 1–15. A further 71 readmissions occurred during days 16–30, such that 241 of 364 readmissions (66.2%) occurred within the first 30 days after discharge. Readmissions during days 31–60 and 61–90 accounted for 78 and 45 events, respectively. Cumulative readmission proportions were 4.0% by day 7, 6.6% by day 15, 9.3% by day 30, and 14.1% by day 90 (Fig. 2).

### GBS was the leading principal diagnosis for 90-day readmissions

**Fig. 3:**
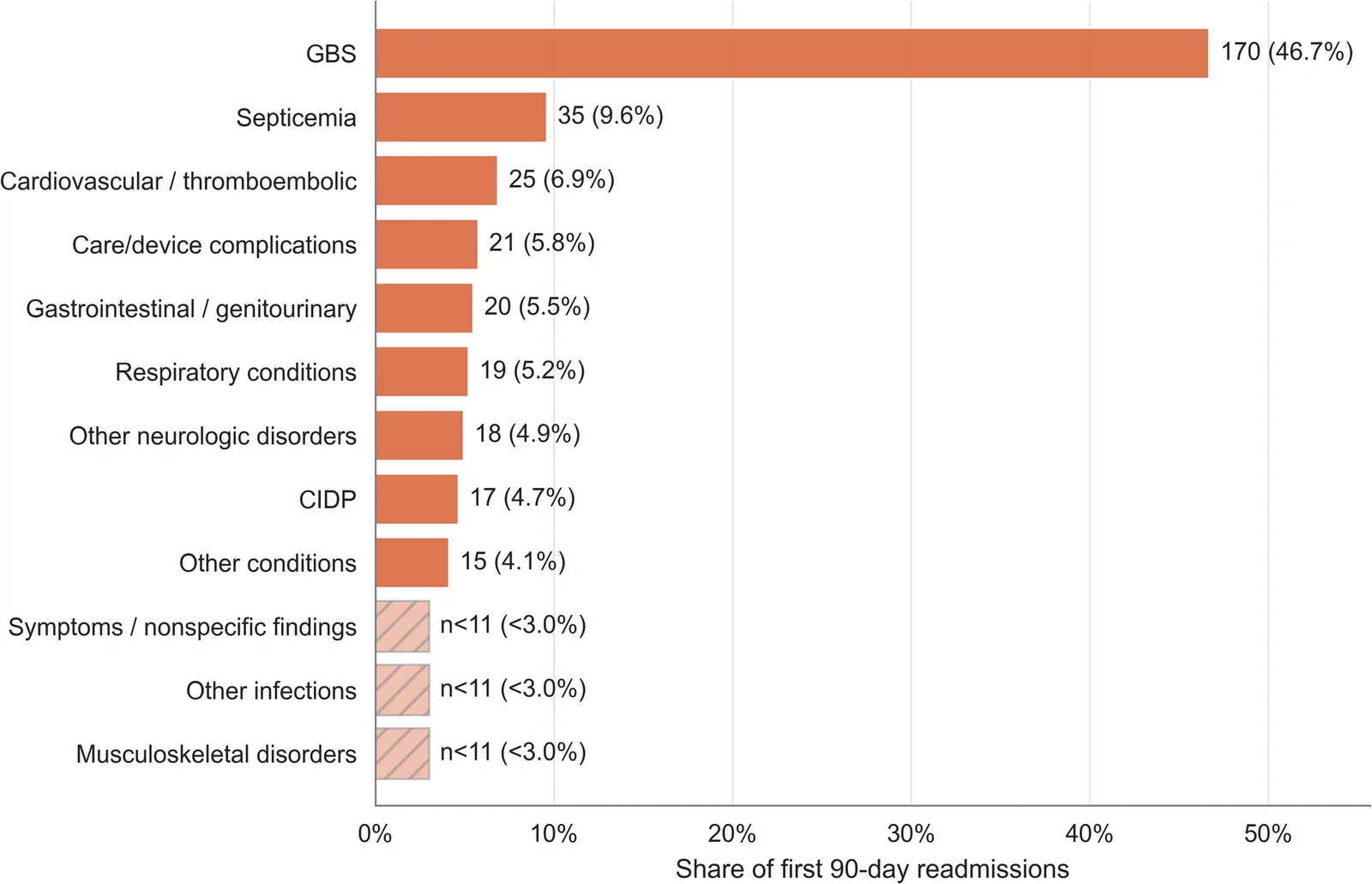
Grouped principal diagnoses of first readmissions within 90 days after index discharge. The analysis included 364 patients with a first readmission within 90 days. Bars show the number and proportion of first readmissions assigned to each grouped principal diagnosis category; percentages were calculated using all 364 first readmissions as the denominator. The polyneuropathy category was further separated by International Classification of Diseases, Tenth Revision, Clinical Modification codes into Guillain-Barré syndrome, chronic inflammatory demyelinating polyradiculoneuropathy, and other neurologic disorders. Nonzero cell counts below 11 are displayed as *n* < 11 and represented by hatched bars in accordance with HCUP reporting requirements. Abbreviations: GBS, Guillain–Barré syndrome; HCUP, Healthcare Cost and Utilization Project

In the separate cause analysis, GBS was the principal diagnosis in 170 of 364 90-day readmissions (46.7%), making it the largest grouped cause of rehospitalization. Among these GBS-coded readmissions, median LOS was 8 days, APR-DRG severity increased compared with the index admission in 42 patients (24.7%), and 12 (7.1%) required mechanical ventilation. In addition, an IVIG code was recorded in 36 patients (21.2%) and PLEX in 40 (23.5%; Suppl. Table 3). This may indicate that a substantial proportion of readmissions were clinically related to the initial GBS episode; however, this interpretation remains speculative because a principal GBS diagnosis cannot distinguish persistent disease, TRF, recurrent GBS, or diagnostic reclassification. Septicemia was the second most frequent principal cause, followed by cardiovascular or thromboembolic conditions, care or device complications, gastrointestinal or genitourinary conditions, respiratory conditions, other neurologic disorders, CIDP, and other conditions (Fig. 3).

### Early and late readmissions showed similar cause profiles

When readmissions were grouped as early (days 1–30) and late (days 31–90), GBS-coded readmissions remained the most frequent cause in both periods. GBS accounted for 122 of 241 (50.6%) early readmissions and 48 of 123 (39.0%) late readmissions. The overall grouped cause distribution did not differ significantly between early and late readmissions in the Monte Carlo permutation chi-square test. GBS-coded readmissions were more common in the early period, but this difference did not remain significant after FDR correction. Thus, no individual grouped cause remained statistically robust (Suppl. Figure 1–2).

### Discharge disposition was the clearest adjusted readmission marker

**Fig. 4:**
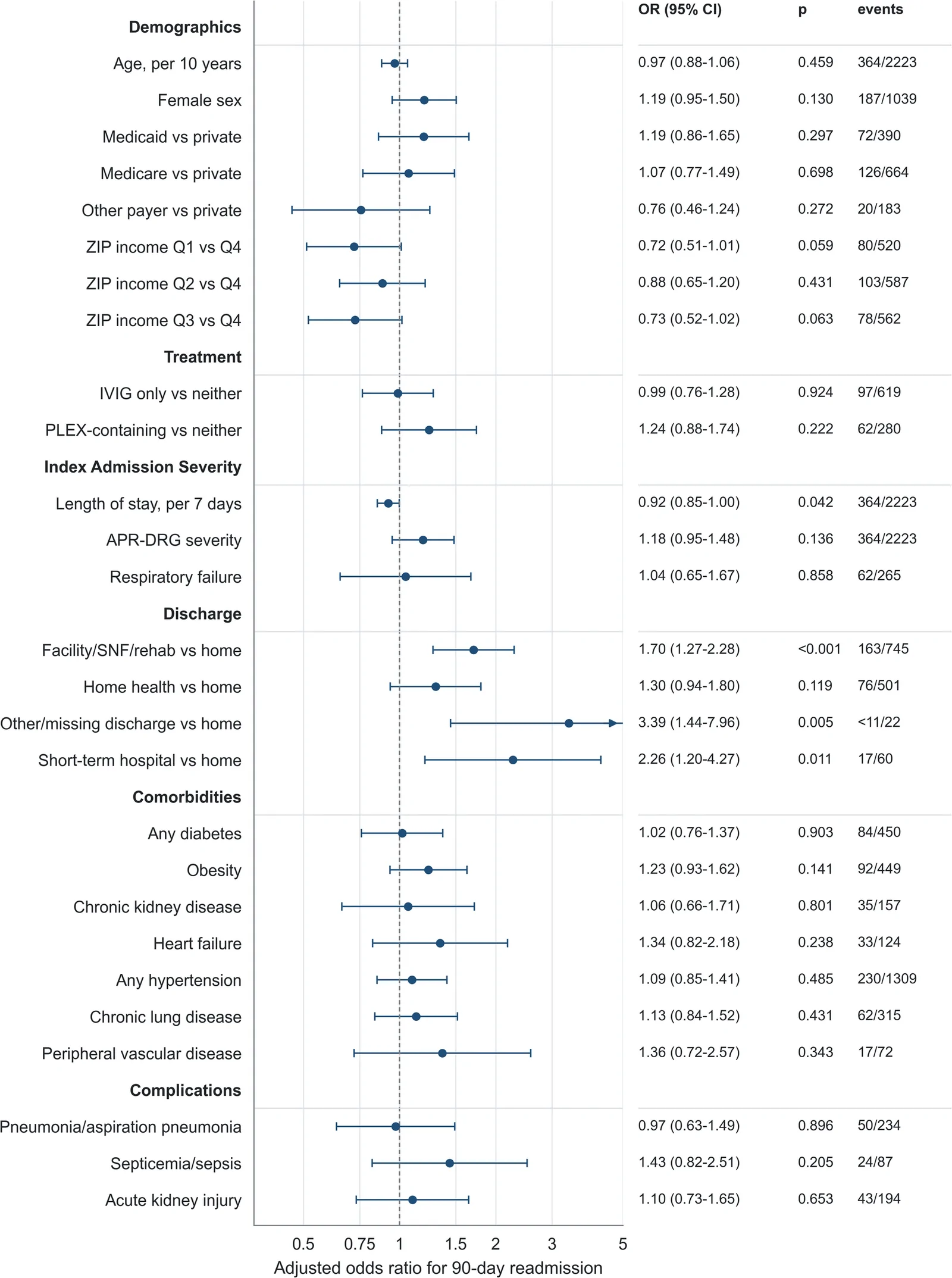
Adjusted associations with first readmission within 90 days after index discharge. A multivariable logistic regression model was fitted in the 90-day eligible GBS cohort (*n* = 2,587), comprising 364 patients with and 2,223 patients without a first 90-day readmission. Points represent adjusted odds ratios, and horizontal lines represent 95% confidence intervals. Confidence intervals and two-sided p-values were calculated using sandwich variance estimates clustered by the hospital of the index admission (846 hospital clusters). The vertical dashed line indicates the null value (OR = 1). Estimates were mutually adjusted for all covariates displayed in the figure. Reference categories were private insurance, the highest ZIP-code income quartile, neither IVIG nor PLEX, and routine home discharge. Values in the right-hand columns report the adjusted OR (95% CI), two-sided p value, and event counts among patients with/without readmission. The x-axis was limited to OR = 5 for readability; arrows indicate confidence intervals extending beyond the displayed range. Reported associations should not be interpreted as causal effects. Abbreviations: APR-DRG, All Patient Refined Diagnosis Related Group; CI, confidence interval; GBS, Guillain–Barré syndrome; IVIG, intravenous immunoglobulin; OR, odds ratio; PLEX, plasma exchange; SNF, skilled nursing facility

In the adjusted 90-day logistic regression model all 2,587 patients were included and no observations were excluded because of missing model covariates. Here, discharge disposition was the clearest readmission marker. Compared with routine home discharge, discharge to a facility, skilled nursing facility, rehabilitation setting, or other non-routine-home destination was associated with higher odds of 90-day readmission (OR 1.70, 95% CI 1.27–2.28). Short-term hospital transfer was also associated with increased odds of readmission (OR 2.26, 95% CI 1.20–4.27). Among patients discharged home, 100 of 995 (10.1%) were readmitted within 90 days, compared with 264 of 1,592 patients (16.6%) discharged to non-routine-home destinations. Index length of stay, scaled per 7 days, showed a weak inverse association with 90-day readmission (OR 0.92, 95% CI 0.85–1.00; *p* = 0.042). LOS alone showed no meaningful discrimination for 90-day readmission (AUROC 0.512, bootstrap 95% CI 0.480–0.542). The data-derived Youden threshold was an LOS of ≥ 12 days, yielding 40.9% sensitivity, 62.6% specificity, 15.2% PPV, and 86.6% NPV. In unadjusted analyses, several treatment-, severity-, and complication-related markers, including PLEX-containing therapy, APR-DRG severity, respiratory failure, obesity, heart failure, and sepsis, were associated with higher readmission odds (Suppl. Figure 3–4), but these associations were attenuated after adjustment. Age, sex, and treatment-code group showed no significant signal (Fig. 4). Excluding treatment category did not materially change the association between discharge disposition and 90-day readmission; discharge to facility/SNF/rehabilitation/other remained associated with readmission (OR 1.70, 95% CI 1.27–2.28; *p* < 0.001). Additionally, an adjustment for procedure-derived markers (supportive care, mechanical ventilation, tracheostomy, gastrostomy, and rehabilitation procedures) minimally changed this association as well (OR 1.65, 95% CI 1.23–2.21). Multicollinearity was not substantial (maximum variance inflation factor, 2.24). Apparent model discrimination was modest (AUROC, 0.624; Brier score, 0.118). The adjusted 30-day model showed a similar disposition-centered pattern (Suppl. Figure 5).

In the timing model comparing early versus late readmission among patients readmitted within 90 days, IVIG-only therapy, short-term hospital transfer, and chronic kidney disease showed nominal associations, however, these findings lost significance after FDR correction (Suppl. Figure 6). Given the restricted sample size, the analysis had limited statistical power and was considered exploratory.

### Procedure timing revealed early immunotherapy and later supportive-care needs

**Fig. 5:**
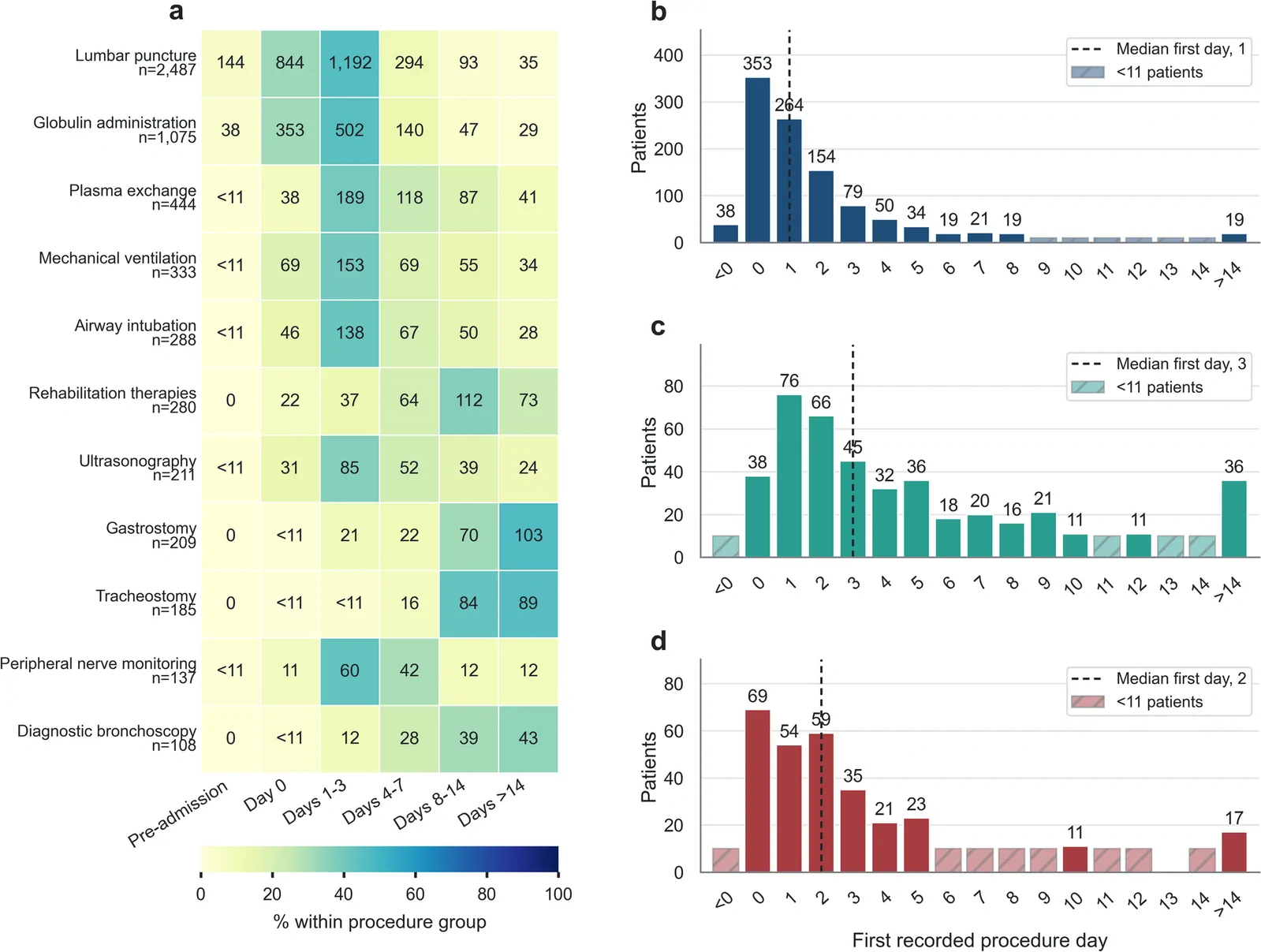
Recorded timing of procedures and therapies relative to the GBS index admission. Recorded procedure timing was determined from ICD-10-PCS procedure-day fields relative to the index admission date. a Row-normalized distribution across pre-admission days − 4 to − 1, admission day 0, days 1–3, 4–7, 8–14, and ≥ 15. Pre-admission procedure days may reflect services performed shortly before formal inpatient admission and incorporated into the subsequent inpatient record; they should not be interpreted as inpatient procedures. In each row, n represents unique patients with available procedure-day information. Cells show patient counts per interval; color intensity reflects within-procedure timing and should not be used to compare absolute frequencies across procedures. Patients may contribute to multiple intervals when procedures were recorded repeatedly. The globulin-administration row represents the IVIG procedure-code proxy, plasma exchange uses the exact procedure-code definition, and remaining rows represent PRCCSR groups. b–d Distribution of each patient’s first recorded procedure day for the IVIG proxy (*n* = 1,075; b), plasma exchange (*n* = 444; c), and mechanical ventilation (*n* = 333; d), thereby avoiding repeated within-patient records. Dashed lines indicate median first recorded procedure day. Pre-admission procedures were combined as < 0 and procedures on day ≥ 15 as > 14. Nonzero counts below 11 are hatched according to HCUP reporting requirements. Administrative procedure-day codes represent recorded coding dates and should not be interpreted as confirmed treatment initiation. Abbreviations: GBS, Guillain–Barré syndrome; HCUP, Healthcare Cost and Utilization Project; ICD-10-PCS, International Classification of Diseases, Tenth Revision, Procedure Coding System; IVIG, intravenous immunoglobulin; PRCCSR, Procedure Clinical Classifications Software Refined

Among all GBS index admissions in 2020 (*n* = 3,548), procedure-code timing during the index hospitalization showed distinct temporal patterns. Lumbar puncture and globulin administration coding clustered on day 0 and days 1–3, while PLEX therapy was shifted later, most commonly appearing during days 1–3 and 4–7. Supportive intensive-care procedures, including mechanical ventilation and airway intubation, clustered around admission. In contrast, rehabilitation therapy, gastrostomy, and tracheostomy showed greater representation in later time bins, reflecting longer or possibly more complex hospitalizations (Fig. 5a). The observed IVIG coding showed 850 of 1,075 first doses (79.1%) administered between admission day 0 and day 3. PLEX showed a later distribution, with 225 of 444 first procedures (50.7%) on days 0–3, but additionally 109 (24.5%) on day 8 or later. Mechanical ventilation was recorded early, with 217 of 333 first recorded events (65.2%) on days 0–3, but retained a later tail beyond day 7 (Fig. 5b-d). In total, 1,444 patients (40.7%) received immunotherapy. Among these, 995 received only IVIG (68.9%), 357 received only PLEX (24.7%), and 92 received both treatments (6.4%).

## Discussion

In this contemporary NRD-derived cohort, GBS was associated with a substantial post-discharge readmission burden. Readmissions occurred early, were most frequently coded as GBS-related, and were accompanied by infection-related causes as secondary contributors. Discharge destination was the most consistent factor associated with readmission. Together, these findings support readmission after GBS as a heterogeneous signal of post-acute vulnerability.

### Readmission burden and early vulnerability

The early temporal clustering of readmissions has important clinical implications. In our cohort, 14.1% of patients were readmitted within 90 days, with nearly half of these readmissions occurring within the first 15 days after discharge and approximately two thirds within the first 30 days. This pattern suggests that the immediate post-discharge period represents a particularly vulnerable period with the need for complication surveillance, rehabilitation coordination, and early reassessment of neurological recovery. Routine home discharge had the lowest observed readmission rate, but it was common and therefore still contributed substantially to the absolute number of readmissions. This distinction is clinically relevant: patients discharged home may deteriorate outside structured programs, whereas patients discharged to facilities or transferred to another hospital appear to carry higher relative vulnerability. Structured follow-up after hospitalization for GBS may therefore be beneficial in identifying relevant deterioration early and initiating timely treatment or supportive interventions. In the future, telemedical approaches and digital monitoring tools could potentially provide scalable strategies to monitor mobility or delayed recovery outside the hospital, as currently studied for other neuromuscular disorders such as CIDP or myasthenia gravis [17–20]. 

Beyond clinical significance, readmissions were also associated with substantial hospital billing amounts. Among readmissions with GBS as the principal diagnosis, median total hospital charges were $98,362. Because these values represent hospital charges rather than actual costs or reimbursements, they should not be interpreted as direct estimates of economic burden. Nevertheless, although lower than charges during the index hospitalization, readmission charges remained substantial and may represent a potentially reducible component of downstream healthcare utilization. The broader burden of rehospitalization may also extend to disruption of rehabilitation trajectories and prolonged dependency, particularly in GBS, where persisting residual weakness, pain, and mobility limitations are common [21, 22]. 

### Readmission causes and complications

Beyond readmissions coded primarily as GBS, septicemia, respiratory complications, and cardiovascular events were prominent secondary causes. Severe cases of GBS may be accompanied by immobility, dysphagia, respiratory muscle weakness, and autonomic dysfunction which may consequently increase the susceptibility to pneumonia, sepsis, and thromboembolic events [6, 23–25]. In general readmission literature, discharge readiness, communication, and follow-up monitoring have been identified as modifiable contributors [26, 27]. Thus, infection- and complication-related readmissions may represent both residual disease severity and potential targets for improved peri-discharge surveillance.

Discharge destination was the strongest factor associated with readmission in our analyses. Compared with routine home discharge, facility discharge and short-term hospital transfer were associated with higher readmission odds. These associations also remained robust after excluding the potentially misclassified procedure-coded treatment variable and adjustment for further severity-markers. However, non-routine-home discharge should not be interpreted as a direct driver of readmission. Rather, discharge destination likely acts as an integrated marker of post-acute vulnerability, reflecting residual neurological disability, medical complexity, and the need for ongoing rehabilitation or nursing support. The similar disposition-centered pattern in our 30-day model supports this interpretation and is consistent with previous studies showing lower readmission risk after routine home discharge when it reflects clinical stability and adequate outpatient support, whereas non-routine-home discharge often identifies patients with greater baseline vulnerability [28–30]. 

Length of stay provided an additional, though less clear, signal. As a standalone predictor, LOS did not meaningfully discriminate between patients with and without readmission; however, in the adjusted regression model, shorter index hospitalization was associated with a higher proportion of 90-day readmission. One possible explanation is that shorter stays may reflect early clinical improvement followed by TRF or renewed deterioration after discharge. Alternatively, shorter hospitalization could indicate discharge before complete stabilization and the appearance of systemic vulnerabilities. In line with this, readmission hospitalizations in our cohort showed higher severity markers than index admissions. However, this interpretation remains speculative, since administrative data do not capture neurological examinations, treatment response, or discharge readiness. Moreover, current literature on LOS is inconclusive: in some conditions, shorter stays have been associated with higher readmission risk [31], whereas in others, longer stays primarily mark greater disease severity and therefore readmission [32, 33]. 

### Prior NRD-based data

Compared with NRD-based data, our findings provide a complementary ICD-10-era perspective. Roberts et al. reported a 12.2% 30-day readmission rate using 2013 NRD and identified respiratory, heart, and renal failure as factors associated with readmission [13]. In our 2020 cohort, the observed 30-day readmission rate was lower at 9.3%, and these complications were not retained as independent markers after adjustment. Although the lower 30-day readmission proportion may suggest temporal change, direct comparison is limited by differences in coding era, cohort construction, and the COVID-19 healthcare context [34] and should therefore be interpreted cautiously. Placed in a broader readmission context, our observed 30-day readmission proportion was below the overall 2020 NRD all-cause readmission proportion reported by HCUP [14], but broadly similar to published estimates in other neuroimmunological conditions with different disease courses such as multiple sclerosis and neuromyelitis optica spectrum disorder [35, 36]. A robust assessment of temporal trends in GBS readmission would require harmonized analyses across multiple NRD years.

### Treatment coding pathways

Recorded index immunotherapy category was not independently associated with readmission in our adjusted models. This is broadly in line with the therapeutic literature, in which IVIG and PLEX remain the established acute immunotherapies showing comparable efficacy. Administrative comparisons, including Beydoun et al. and Roberts et al., have suggested possible outcome differences between PLEX and IVIG, but these analyses remain vulnerable to confounding by indication [13, 37]. 

Notably, the proportion of patients with coded immunotherapy (40.7%) was lower than in prospective GBS cohorts [38], but comparable to U.S. administrative datasets [37, 39]. This may reflect both incomplete IVIG capture and genuinely untreated mild cases [7]. 

Current EAN/PNS guidelines recommend IVIG or PLEX within appropriate time windows and advise against PLEX followed immediately by IVIG or vice versa [7]. Thus, despite established acute immunotherapies, treatment options remain limited, and a substantial proportion of patients still experience prolonged hospital courses and intensive care procedures. In this context, the procedure-coding heatmap provides a descriptive view of real-world coding patterns: diagnostic evaluation and early immunotherapy seem to be performed early, while escalation, respiratory support, nutritional access, and rehabilitation needs emerge later in longer or more complex hospital stays. Importantly, the later PLEX distribution may partly reflect procedural logistics rather than treatment sequencing.

Overall, this coding sequence underscores that GBS management extends well beyond the initial immunotherapy decision. It requires prolonged, resource-intensive treatment escalation and complication management. Therefore, future advances in GBS should combine earlier diagnostic certainty, improved risk stratification, and more effective therapies to reduce late escalation, prolonged intensive-care need, and discharge disability.

### Strengths and limitations

A key strength of this study is its real-world perspective. Using an ICD-10-era NRD-derived cohort from 2020, it captures a broad range of hospital-based cases across the US rather than a highly selected prospective cohort. The combination of readmission timing and procedure-code-based pathway visualization adds insight into early post-discharge vulnerability and acute care patterns beyond readmission rates alone. However, several limitations should be considered. Analyses were performed unweighted; therefore, the results describe the available NRD-derived cohort rather than weighted national estimates. In addition, all definitions relied on administrative ICD-10-CM/PCS coding without chart adjudication. Consequently, GBS-coded readmissions could not be differentiated into persistent disability, TRF, recurrent GBS or evolving CIDP. Also, the 90-day analysis was restricted to January–September discharges. Comparison of baseline characteristics with patients discharged from October–December suggested that the primary cohort may slightly underrepresent older or more complex patients hospitalized later in 2020. In this context, out-of-hospital mortality and readmissions not captured within the NRD may additionally affect readmission proportion. Furthermore, IVIG capture relied on specific globulin-administration procedure codes and may therefore underestimate true IVIG use. Finally, the 2020 study year was shaped by pandemic-related changes. Compared with 2019, overall NRD discharges declined, particularly during the second quarter, while the overall 30-day readmission rate remained largely stable [40]. Changes in healthcare utilization and rehabilitation may nevertheless have influenced LOS, treatment, and readmission patterns, and results should be interpreted within the COVID-era context.

## Conclusions

Readmission after GBS remains a frequent and clinically relevant outcome. Its early clustering, heterogeneous causes, and strong association with discharge destination suggest that readmission reflects residual neurological disability, systemic complications, and care-transition vulnerability rather than recurrent GBS alone. Improving post-discharge surveillance, rehabilitation continuity, and future treatment strategies may therefore be important for reducing the downstream burden of GBS.

## Supplementary Information

Below is the link to the electronic supplementary material.

Supplementary Material 1

## Data Availability

The data used in this study were obtained from the Healthcare Cost and Utilization Project (HCUP) Nationwide Readmissions Database (NRD). Due to the HCUP Data Use Agreement, the underlying patient-level data cannot be shared by the authors. The NRD is available from HCUP/AHRQ to eligible researchers after completion of the required data use training and execution of a data use agreement.
