# Supplementary material for: Real-world hospitalization and readmission trajectory after Guillain-Barré syndrome in the United States
Source: BMC Neurol. 2026 Oct 22;26:702. doi: 10.1186/s12883-026-05499-9 (PMC13733053; doi:10.1186/s12883-026-05499-9)
Supplement: Supplementary file 1 [file 12883_2026_5499_MOESM1_ESM.docx]

**Supplementary material**

Table of Contents

[Supplementary Table 1	3](#_Toc239167605)

[Supplementary Table 2	5](#_Toc239167606)

[Supplementary Table 3	6](#_Toc239167607)

[Supplementary Table 4	7](#_Toc239167608)

[Supplementary Figure 1	9](#_Toc239167609)

[Supplementary Figure 2	10](#_Toc239167610)

[Supplementary Figure 3	11](#_Toc239167611)

[Supplementary Figure 4	12](#_Toc239167612)

[Supplementary Figure 5	13](#_Toc239167613)

[Supplementary Figure 6	14](#_Toc239167614)

# **Supplementary Table 1**

| **Characteristic** | **Admission cohort (N=3,548)** | **Missing n** |
| --- | --- | --- |
| Age, years | 56.0 [40.0-67.0] | 0 |
| Female sex | 1,698 (47.9%) | 0 |
| **Primary expected payer** |  | 4 |
| Private | 1,490 (42.0%) |  |
| Medicare | 1,144 (32.2%) |  |
| Medicaid | 646 (18.2%) |  |
| Other | 268 (7.6%) |  |
| **ZIP income quartile** |  | 0 |
| Q1 lowest | 846 (23.8%) |  |
| Q2 | 958 (27.0%) |  |
| Q3 | 878 (24.7%) |  |
| Q4 highest | 866 (24.4%) |  |
| **Patient residence (NCHS urban-rural)** |  | 9 |
| Central metro >=1 million | 944 (26.6%) |  |
| Fringe metro >=1 million | 931 (26.2%) |  |
| Metro 250,000-999,999 | 795 (22.4%) |  |
| Metro 50,000-249,999 | 338 (9.5%) |  |
| Micropolitan | 316 (8.9%) |  |
| Noncore | 215 (6.1%) |  |
| **Hospital bed size** |  | 0 |
| Small | 458 (12.9%) |  |
| Medium | 903 (25.5%) |  |
| Large | 2,187 (61.6%) |  |
| **Hospital location/teaching status** |  | 0 |
| Non-metropolitan | 588 (16.6%) |  |
| Metropolitan non-teaching | 2,862 (80.7%) |  |
| Metropolitan teaching | 98 (2.8%) |  |
| **Hospital urban-rural category** |  | 0 |
| Large metropolitan | 2,072 (58.4%) |  |
| Small metropolitan | 1,378 (38.8%) |  |
| Micropolitan | 91 (2.6%) |  |
| Nonurban residual | <11 (<0.3%) |  |
| **APR-DRG severity of illness** |  | 0 |
| Minor | <11 (0.3%) |  |
| Moderate | 2,268 (63.9%) |  |
| Major | 782 (22.0%) |  |
| Extreme | 493 (13.9%) |  |
| Index length of stay, days | 9.0 [5.0-19.0] | 0 |
| Index admission total charges, $ | $143,970 [$87,948-$243,786] | 33 |
| **Index immunotherapy** |  | 0 |
| Neither IVIG nor PLEX | 2,104 (59.3%) |  |
| IVIG only | 995 (28.0%) |  |
| PLEX-containing therapy | 449 (12.7%) |  |
| IVIG and PLEX | 92 (2.6%) |  |
| PLEX only | 357 (10.1%) |  |
| **Respiratory Complications** |  | 0 |
| Mechanical ventilation | 342 (9.6%) |  |
| Respiratory failure | 469 (13.2%) |  |
| **Discharge disposition** |  | 1 |
| Routine home | 1,316 (37.1%) |  |
| Short-term hospital transfer | 110 (3.1%) |  |
| Home health care | 795 (22.4%) |  |
| Facility / SNF / rehab / other | 1,284 (36.2%) |  |
| Other / missing | 43 (1.2%) |  |
| **Comorbidities** |  | 0 |
| Any diabetes | 779 (22.0%) |  |
| Obesity | 746 (21.0%) |  |
| Chronic kidney disease | 279 (7.9%) |  |
| Heart failure | 223 (6.3%) |  |
| Any hypertension | 2,130 (60.0%) |  |
| Chronic lung disease | 529 (14.9%) |  |
| Peripheral vascular disease | 115 (3.2%) |  |
| Pneumonia/aspiration pneumonia | 404 (11.4%) |  |
| Septicemia/sepsis | 151 (4.3%) |  |
| Acute kidney injury | 371 (10.5%) |  |

**Suppl. Table 1. Baseline demographic, clinical, and hospital characteristics of the GBS index-admission cohort**

Data are presented as median [interquartile range] for continuous variables and n (%) for categorical variables. Percentages were calculated using nonmissing observations. Hospital bed size was classified as small, medium, or large according to HCUP-NRD definitions accounting for hospital region, location, and teaching status. Cell counts below 11 were suppressed in accordance with HCUP reporting requirements.

Abbreviations: APR-DRG, All Patient Refined Diagnosis Related Group; GBS, Guillain–Barré syndrome; HCUP, Healthcare Cost and Utilization Project; IVIG, intravenous immunoglobulin; NRD, Nationwide Readmissions Database; PLEX, plasma exchange; SNF, skilled nursing facility.

# **Supplementary Table 2**

| **Measure** | **Paired N** | **Index admission** | **First 90-day readmission** | **Wilcoxon p value** | **Paired change, median [IQR]** | **Decreased, n (%)** | **Unchanged, n (%)** | **Increased, n (%)** |
| --- | --- | --- | --- | --- | --- | --- | --- | --- |
| Length of stay, days | 364 | 9.00 [5.00-18.00] | 8.00 [4.00-16.00] | 0.006 | -1.00 [-7.00-4.00] | 203 (55.77%) | 19 (5.22%) | 142 (39.01%) |
| APR-DRG severity score | 364 | 2.00 [2.00-3.00] | 3.00 [2.00-4.00] | <0.001 | 0.00 [0.00-1.00] | 62 (17.03%) | 180 (49.45%) | 122 (33.52%) |
| APR-DRG mortality risk score | 364 | 1.00 [1.00-2.00] | 2.00 [1.00-3.00] | <0.001 | 0.00 [0.00-1.00] | 45 (12.36%) | 185 (50.82%) | 134 (36.81%) |
| Mechanical ventilation | 364 | 47 (12.91%) | 56 (15.38%) | 0.24 | 0.00 [0.00-0.00] | 25 (6.87%) | 305 (83.79%) | 34 (9.34%) |

**Suppl. Table 2. Paired index and readmission severity characteristics**

Values are shown among patients with an eligible first 90-day readmission in the finalized GBS-only cohort. Continuous, ordinal, and charge measures are summarized as median [IQR]; binary measures are summarized as n (%). Change is readmission value minus index value. Direction columns report n (%) using the paired denominator for each measure; nonzero event counts below 11 are suppressed as n<11.

Abbreviations: APR-DRG, All Patient Refined Diagnosis Related Group; GBS, Guillain-Barre syndrome; IQR, interquartile range; LOS, length of stay.

# **Supplementary Table 3**

| **Readmission cause group** | **N** | **Index LOS, median (days)** | **Readmission LOS, median (days)** | **LOS difference, median (days)** | **Longer LOS, n (%)** | **APR-DRG severity increased, n (%)** | **Mechanical ventilation, n (%)** | **IVIG proxy, n (%)** | **PLEX, n (%)** | **Total charges, median [IQR], $** |
| --- | --- | --- | --- | --- | --- | --- | --- | --- | --- | --- |
| GBS | 170 | 6.00 | 8.00 | 0.00 | 78 (45.88%) | 42 (24.71%) | 12 (7.06%) | 36 (21.18%) | 40 (23.53%) | 98,362 [60,170–174,993] |
| Septicemia | 35 | 12.00 | 11.00 | -5.00 | 13 (37.14%) | 18 (51.43%) | 20 (57.14%) | n<11 | n<11 | 104,856 [57,054–204,661] |
| Cardiovascular / thromboembolic | 25 | 8.00 | 4.00 | -6.00 | n<11 | n<11 | n<11 | n<11 | 0 (0.00%) | 42,963 [35,320–89,149] |
| Care/device complications | 21 | 8.00 | 5.00 | -2.00 | n<11 | n<11 | n<11 | 0 (0.00%) | 0 (0.00%) | 53,107 [26,848–152,901] |
| Gastrointestinal / genitourinary | 20 | 11.00 | 5.00 | -4.00 | n<11 | n<11 | n<11 | 0 (0.00%) | n<11 | 46,300 [28,733–82,709] |
| Respiratory conditions | 19 | 20.00 | 10.00 | -11.00 | n<11 | n<11 | 11 (57.89%) | 0 (0.00%) | n<11 | 138,960 [87,234–278,902] |
| Other neurologic disorders | 18 | 7.50 | 10.00 | 0.50 | n<11 | n<11 | n<11 | n<11 | n<11 | 131,200 [68,846–215,957] |
| CIDP | 17 | 8.00 | 10.00 | 0.00 | n<11 | n<11 | n<11 | n<11 | n<11 | 107,529 [78,632–185,695] |
| Other conditions | 15 | 12.00 | 7.00 | -4.00 | n<11 | n<11 | 0 (0.00%) | 0 (0.00%) | n<11 | 93,306 [57,476–152,394] |
| Musculoskeletal disorders | n<11 |  |  |  |  |  |  |  |  |  |
| Other infections | n<11 |  |  |  |  |  |  |  |  |  |
| Symptoms / nonspecific findings | n<11 |  |  |  |  |  |  |  |  |  |

**Suppl. Table 3. Severity characteristics by readmission cause group**

Values are shown among patients with an eligible first 90-day readmission in the finalized GBS-only cohort. LOS difference is readmission LOS minus index LOS. Event summaries are shown as n (%) using the cause-group denominator. IVIG was identified using the globulin-administration procedure-code proxy; PLEX was identified using prespecified ICD-10-PCS codes. Total charges were obtained from the NRD TOTCHG variable and represent hospital billing charges rather than actual costs or reimbursed amounts.Cause groups and nonzero event counts below 11 are suppressed as n<11; corresponding percentages are not shown.

Abbreviations: APR-DRG, All Patient Refined Diagnosis Related Group; GBS, Guillain-Barre syndrome; IVIG, intravenous immunoglobulin; LOS, length of stay; PLEX, plasma exchange; IQR, interquartile range.

# **Supplementary Table 4**

| **Characteristic** | **Included: Jan-Sep (n=2,587)** | **Excluded: Oct-Dec (n=961)** | **Absolute SMD** |
| --- | --- | --- | --- |
| **Demographic and socioeconomic characteristics** | | | |
| Age, years | 55.0 [40.0-66.0] | 58.0 [41.0-69.0] | **0.139** |
| Female sex | 1,226 (47.4%) | 472 (49.1%) | 0.035 |
| **Primary expected payer** |  |  |  |
| Private | 1,132 (43.8%) | 358 (37.3%) | **0.133** |
| Medicare | 790 (30.6%) | 354 (36.9%) | **0.134** |
| Medicaid | 462 (17.9%) | 184 (19.2%) | 0.033 |
| Other | 200 (7.7%) | 64 (6.7%) | 0.042 |
| *Missing* | *<11* | *<11* |  |
| **ZIP income quartile** |  |  |  |
| Q1 lowest | 600 (23.5%) | 246 (26.0%) | 0.057 |
| Q2 | 656 (25.7%) | 254 (26.8%) | 0.026 |
| Q3 | 640 (25.1%) | 238 (25.1%) | 0.001 |
| Q4 highest | 657 (25.7%) | 209 (22.1%) | 0.086 |
| **Patient residence (NCHS urban-rural)** |  |  |  |
| Central metro ≥1 million | 692 (26.8%) | 252 (26.3%) | 0.012 |
| Fringe metro ≥1 million | 700 (27.1%) | 231 (24.1%) | 0.070 |
| Metro 250,000-999,999 | 560 (21.7%) | 235 (24.5%) | 0.066 |
| Metro 50,000-249,999 | 245 (9.5%) | 93 (9.7%) | 0.007 |
| Micropolitan | 232 (9.0%) | 84 (8.8%) | 0.008 |
| Noncore | 151 (5.9%) | 64 (6.7%) | 0.034 |
| *Missing* | *<11* | *<11* |  |
| **Hospital and index-admission characteristics** | | | |
| **Hospital bed size** |  |  |  |
| Small | 327 (12.6%) | 131 (13.6%) | 0.029 |
| Medium | 657 (25.4%) | 246 (25.6%) | 0.005 |
| Large | 1,603 (62.0%) | 584 (60.8%) | 0.025 |
| **Hospital location/teaching status** |  |  |  |
| Non-metropolitan | 424 (16.4%) | 164 (17.1%) | 0.018 |
| Metropolitan non-teaching | 2,095 (81.0%) | 767 (79.8%) | 0.029 |
| Metropolitan teaching | 68 (2.6%) | 30 (3.1%) | 0.030 |
| **Hospital urban-rural category** |  |  |  |
| Large metropolitan | 1,538 (59.5%) | 534 (55.6%) | 0.079 |
| Small metropolitan | 981 (37.9%) | 397 (41.3%) | 0.069 |
| Micropolitan | 61 (2.4%) | 30 (3.1%) | 0.047 |
| Nonurban residual | <11 | 0 (0.0%) | 0.074 |
| **APR-DRG severity of illness** |  |  |  |
| Minor | <11 | 0 (0.0%) | 0.062 |
| Moderate | 1,688 (65.2%) | 580 (60.4%) | **0.101** |
| Major | 558 (21.6%) | 224 (23.3%) | 0.042 |
| Extreme | 336 (13.0%) | 157 (16.3%) | 0.095 |
| Index length of stay, days | 8.0 [5.0-18.0] | 10.0 [5.0-20.0] | 0.074 |
| Index admission total charges, $ | $140,622 [$87,248-$239,194] | $152,665 [$90,763-$263,166] | 0.087 |
| *Missing* | *24 (0.9%)* | *<11* |  |
| **Index immunotherapy** |  |  |  |
| Neither IVIG nor PLEX | 1,529 (59.1%) | 575 (59.8%) | 0.015 |
| IVIG only | 716 (27.7%) | 279 (29.0%) | 0.030 |
| PLEX-containing therapy | 342 (13.2%) | 107 (11.1%) | 0.064 |
| Mechanical ventilation | 251 (9.7%) | 91 (9.5%) | 0.008 |
| Respiratory failure | 327 (12.6%) | 142 (14.8%) | 0.062 |
| **Discharge disposition** |  |  |  |
| Routine home | 995 (38.5%) | 321 (33.4%) | **0.106** |
| Short-term hospital transfer | 77 (3.0%) | 33 (3.4%) | 0.026 |
| Home health care | 577 (22.3%) | 218 (22.7%) | 0.009 |
| Facility / SNF / rehabilitation / other | 908 (35.1%) | 376 (39.1%) | 0.083 |
| Other discharge | 29 (1.1%) | 13 (1.4%) | 0.021 |
| *Missing* | *<11* | *0 (0.0%)* |  |
| **Comorbidities** | | | |
| Any diabetes | 534 (20.6%) | 245 (25.5%) | **0.115** |
| Obesity | 541 (20.9%) | 205 (21.3%) | 0.010 |
| Chronic kidney disease | 192 (7.4%) | 87 (9.1%) | 0.059 |
| Heart failure | 157 (6.1%) | 66 (6.9%) | 0.032 |
| Any hypertension | 1,539 (59.5%) | 591 (61.5%) | 0.041 |
| Chronic lung disease | 377 (14.6%) | 152 (15.8%) | 0.035 |
| Peripheral vascular disease | 89 (3.4%) | 26 (2.7%) | 0.043 |
| **Index-admission complications** | | | |
| Pneumonia/aspiration pneumonia | 284 (11.0%) | 120 (12.5%) | 0.047 |
| Septicemia/sepsis | 111 (4.3%) | 40 (4.2%) | 0.006 |
| Acute kidney injury | 237 (9.2%) | 134 (13.9%) | **0.150** |

**Supplementary Table 4. Baseline characteristics of patients included in and excluded from the primary 90-day analysis**

Values are median [interquartile range] for continuous variables and n (%) for categorical variables. Percentages use nonmissing observations as the denominator. Missingness is reported when present. Absolute standardized mean differences (SMDs) quantify balance; values ≥0.10 indicate potentially meaningful imbalance. For multicategory variables, SMDs are reported for each level. Counts from 1 to 10 are suppressed as <11 in accordance with HCUP reporting requirements. The October-December group was excluded because complete 90-day follow-up was unavailable within the 2020 calendar-year NRD; 90-day outcomes were therefore not compared between groups.

**Abbreviations.** APR-DRG, All Patient Refined Diagnosis Related Group; GBS, Guillain-Barré syndrome; HCUP, Healthcare Cost and Utilization Project; IVIG, intravenous immunoglobulin; NCHS, National Center for Health Statistics; NRD, Nationwide Readmissions Database; PLEX, plasma exchange; SMD, standardized mean difference; SNF, skilled nursing facility.

# **Supplementary Figure 1**

**Supplementary Fig. 1. Grouped principal diagnosis categories of first readmission by timing interval.**

Grouped principal diagnosis categories are shown for the 90-day readmission cohort (n=364), stratified into first readmissions occurring 1–30 days after index discharge (n = 241) and 31–90 days after index discharge (n = 123). **a** First readmissions occurring 1–30 days after index discharge. **b** First readmissions occurring 31–90 days after index discharge. Bars show the percentage of readmissions within each timing interval. Nonzero categories with cell counts of 10 or fewer were masked and displayed as n<11 in accordance with Healthcare Cost and Utilization Project reporting requirements.

Abbreviations: CIDP, chronic inflammatory demyelinating polyradiculoneuropathy; GBS, Guillain-Barré syndrome; HCUP, Healthcare Cost and Utilization Project.

# **Supplementary Figure 2**

**Supplementary Fig. 2.** **Comparison of grouped principal readmission diagnosis categories between early and later readmission.**

Grouped principal diagnosis categories are shown for the 90-day readmission cohort (n=364), stratified into first readmissions occurring 1–30 days after index discharge (n = 241) and 31–90 days after index discharge (n = 123). Bars show the percentage-point difference between readmissions occurring 1–30 days and 31–90 days after discharge, calculated as early minus later; positive values therefore indicate greater representation among early readmissions. The right column reports early and later counts, category-specific odds ratios with 95% confidence intervals, two-sided Fisher exact p values, and Benjamini-Hochberg false-discovery-rate adjusted q values. The global Monte Carlo permutation chi-square test for the grouped distribution was p=0.210. Category-level comparisons were exploratory, and sparse cells were masked according to Healthcare Cost and Utilization Project reporting requirements.

Abbreviations: CI, confidence interval; FDR, false discovery rate; GBS, Guillain-Barré syndrome; HCUP, Healthcare Cost and Utilization Project; OR, odds ratio.

# **Supplementary Figure 3**

**Supplementary Fig. 3. Unadjusted associations with first readmission within 90 days after index discharge.**

Each row represents a separate univariable logistic regression model fitted in the 90-day eligible GBS cohort (n=2,587), comprising 364 patients with and 2,223 patients without a first 90-day readmission. The outcome was the first readmission occurring 1–90 days after index discharge. Points represent unadjusted odds ratios (ORs), and horizontal lines represent 95% confidence intervals (CIs). Confidence intervals and two-sided p-values were calculated using sandwich variance estimates clustered by the hospital of the index admission (846 hospital clusters). The vertical dashed line indicates the null value (OR=1). Categorical estimates are relative to the reference categories shown. Values in the right-hand columns report the OR (95% CI), two-sided p value, and event counts among exposed and unexposed patients. Estimates were not adjusted for other covariates.

Abbreviations: APR-DRG, All Patient Refined Diagnosis Related Group; CI, confidence interval; IVIG, intravenous immunoglobulin; OR, odds ratio; PLEX, plasma exchange.

# **Supplementary Figure 4**

**Supplementary Fig. 4. Unadjusted associations with first readmission within 30 days after index discharge.**

Each row represents a separate univariable logistic regression model fitted in the 30-day eligible GBS cohort (n=3,221), comprising 298 patients with and 2,923 patients without a first 30-day readmission. The outcome was the first readmission occurring 1–30 days after index discharge. Points represent unadjusted odds ratios (ORs), and horizontal lines represent 95% confidence intervals (CIs). Confidence intervals and two-sided p-values were calculated using sandwich variance estimates clustered by the hospital of the index admission (922 hospital clusters). The vertical dashed line indicates the null value (OR=1). Categorical estimates are relative to the reference categories shown. Values in the right-hand columns report the OR (95% CI), two-sided p value, and event counts among exposed and unexposed patients. Estimates were not adjusted for other covariates.

Abbreviations: APR-DRG, All Patient Refined Diagnosis Related Group; CI, confidence interval; IVIG, intravenous immunoglobulin; OR, odds ratio; PLEX, plasma exchange.

# **Supplementary Figure 5**

**Supplementary Fig. 5. Adjusted associations with first readmission within 30 days after index discharge.**

The multivariable logistic regression model was fitted in the 30-day eligible GBS cohort (complete-case n=3,221), comprising 298 patients with and 2,923 patients without a first 30-day readmission. The outcome was the first readmission occurring 1–30 days after index discharge. Points represent adjusted odds ratios (ORs), and horizontal lines represent 95% confidence intervals (CIs). The vertical dashed line indicates the null value (OR=1). Estimates were mutually adjusted for the covariates displayed in the figure. Categorical estimates are relative to the reference categories shown. Values in the right-hand columns report the adjusted OR (95% CI). Confidence intervals and two-sided p-values were calculated using sandwich variance estimates clustered by the hospital of the index admission (922 hospital clusters). Event counts show exposed and unexposed patients.

Abbreviations: APR-DRG, All Patient Refined Diagnosis Related Group; CI, confidence interval; IVIG, intravenous immunoglobulin; OR, odds ratio; PLEX, plasma exchange.

# **Supplementary Figure 6**

**Supplementary Fig. 6. Adjusted associations with earlier versus later readmission among patients readmitted within 90 days.**

The multivariable logistic regression model included patients with a first readmission within 90 days after index discharge (n=364), comprising 241 readmissions at 1–30 days and 123 at 31–90 days. The outcome contrasted earlier (1–30 days) with later (31–90 days) readmission; therefore, ORs greater than 1 indicate higher odds of earlier readmission. Points represent adjusted ORs, and horizontal lines represent 95% CIs. The vertical dashed line indicates OR=1. Estimates were mutually adjusted for the covariates displayed, and categorical estimates are relative to the reference categories shown. Two-sided p values and Benjamini–Hochberg false-discovery-rate-adjusted q values are reported. Arrows indicate confidence intervals extending beyond the displayed x-axis.

Abbreviations: APR-DRG, All Patient Refined Diagnosis Related Group; CI, confidence interval; IVIG, intravenous immunoglobulin; OR, odds ratio; PLEX, plasma exchange.
